# Hypocarnitinemia and its effect on seizure control in adult patients with intractable epilepsy on the modified Atkins diet

**DOI:** 10.3389/fnut.2023.1304209

**Published:** 2024-01-05

**Authors:** Daniel Y. Chu, Michele N. Ravelli, Kelly M. Faltersack, Arron L. Woods, Dace Almane, Zhanhai Li, Emmanuel Sampene, Elizabeth A. Felton

**Affiliations:** ^1^Department of Neurology, University of Wisconsin School of Medicine and Public Health, Madison, WI, United States; ^2^Department of Radiology, University of Wisconsin School of Medicine and Public Health, Madison, WI, United States; ^3^Department of Clinical Nutrition, University of Wisconsin Hospitals and Clinics, Madison, WI, United States; ^4^Department of Biostatistics and Medical Informatics, University of Wisconsin School of Medicine and Public Health, Madison, WI, United States

**Keywords:** modified Atkins diet, ketogenic therapy, seizure, epilepsy, carnitine, ketogenic dietary therapy

## Abstract

**Introduction:**

Previous studies have demonstrated the safety and efficacy of the modified Atkins diet (MAD) in attenuating seizures in patients with intractable epilepsy. MAD works by achieving ketosis, which is heavily dependent on the metabolic compound, carnitine, to facilitate the transport of long-chain fatty acids across the mitochondria for beta-oxidation. The effect of carnitine on ketogenic diet therapy is not well-defined in the current literature. Thus, the purpose of our study is to investigate the effects of hypocarnitinemia on the efficacy of MAD.

**Methods:**

A retrospective chart review was conducted, and 58 adults with epilepsy undergoing MAD were evaluated. Generalized linear mixed effects models were used to compare the low carnitine status with normal carnitine group in patient measures of body mass index, seizure frequency and severity, number of anti-seizure medications, beta-hydroxybutyrate, triglyceride, and carnitine levels across baseline, 3–9-month follow-up (timepoint 1), 1-2-year follow-up (timepoint 2), and 2+ year follow-up (timepoint 3).

**Results:**

Our study revealed that 38.3% of adult patients with epilepsy following MAD experienced low free carnitine at some point through the course of diet therapy. Patients with hypocarnitinemia at timepoint 2 showed a significant percent seizure increase while seizures continued to decrease in the normal carnitine group. Fasting triglyceride levels at timepoint 1 were significantly increased in the low carnitine group compared to normal carnitine group. Change in BHB, BMI, seizure severity, and number of ASMs showcased no significant differences between the low and normal carnitine groups.

**Discussion:**

It may be important for clinicians to monitor for hypocarnitinemia in adults on MAD and provide carnitine supplementation when low. Further investigations into carnitine and MAD may inform clinical decisions on carnitine supplementation to maximize the efficacy of MAD therapy.

## 1 Introduction

Epilepsy is the 4th most common neurological disorder, with a prevalence of over 2.2 million in the United States and 65 million worldwide (1–3). Current treatments include anti-seizure medications (ASM), surgical interventions, neurostimulation, and ketogenic diet therapy. Despite the myriad of ASMs available, 1/3 of the patients are considered drug-resistant or refractory (intractable epilepsy) (4, 5). As a result, the modified Atkins diet (MAD), a form of ketogenic diet therapy that is high in fat and low in net carbohydrates at no more than 10–20 g per day, has been increasingly explored as an adjunctive treatment for adult patients with intractable epilepsy (6). The positive effects of the diet are attributed to maintaining a state of nutritional ketosis, which is also a marker for compliance to the diet and assessed by elevated levels of ketone bodies such as acetoacetate in the urine, beta-hydroxybutyrate (BHB) in the blood, and acetone in the breath (7, 8). MAD has several advantages including that it is safe, effective, can be rapidly initiated, and that its beneficial effects can be observed almost immediately (7). Several previous studies have demonstrated the efficacy of MAD in its seizure reduction potential in both the adult and pediatric populations (9–12).

To achieve nutritional ketosis on MAD, restrictive carbohydrate and moderate protein intake coupled with a high fat consumption is necessary (6, 9). Ketogenesis depends on the transport of long-chain fatty acids, which contain 13 to 21 carbons, into the mitochondria for beta-oxidation (13, 14). The critical metabolic compound responsible for facilitating this process is carnitine, a quaternary ammonium compound biologically active in its L isoform (13, 15, 16). Carnitine acts as a co-factor and esterifies the long-chain fatty acid for its transport across the mitochondria (13). The status of carnitine is evaluated by measuring plasma levels of total carnitine, free carnitine, and acyl-carnitine (17). Because carnitine is responsible for this crucial step in the generation of ketones, MAD will likely increase demand for carnitine. In theory, low carnitine or hypocarnitinemia should disrupt the ketone generation metabolic pathway, affecting the efficacy of this diet therapy in combatting seizures (18). Although previous research has shown MAD to be highly effective in attenuating seizures, few studies have evaluated whether low plasma free carnitine plays a role in the patient’s state of ketosis while on the diet.

The majority of carnitine studies have focused on the pediatric population. In recent literature, Berry-Kravis et al. (15) examined whether the ketogenic diet can induce carnitine deficiency due to high fat content and reported that 6 of their 46 (8.7%) patients initially developed low carnitine levels in the first 6 months. Previous studies have also reported carnitine deficiency in patients taking valproic acid and having a multiple ASM regimen (13, 15, 19). Coppola et al. (13) evaluated carnitine levels in children with or without ketogenic diet. In their study of patients who are not on the diet, carnitine deficiency was found in 32 out of their 84 (38%) patients taking valproic acid and 13 out of 54 (24%) taking carbamazepine, both as monotherapy and utilized in combination with other ASMs. In addition, they found that lower carnitine levels were associated with polytherapy as opposed to monotherapy, but the results were not statistically significant. However, they reported that none of their 11 patients with epilepsy on the ketogenic diet developed abnormal levels of free carnitine. In adults on modified ketogenic diets, Roehl et al. (12) described that 8 out of 26 (31%) patients showed carnitine deficiency at baseline and 2 out of 7 (29%) patients showed carnitine deficiency at 3-month follow up. Despite this, they did not investigate how hypocarnitinemia impacted the efficacy of diet therapy. Therefore, the direct relationship between carnitine and its effect on ketogenic diet therapy in the adult population is still not well-defined in current literature.

For patients undergoing MAD, carnitine levels are initially evaluated at baseline (prior to starting ketogenic therapy). In pediatrics, carnitine levels are routinely checked at follow ups; however, in adults, carnitine levels may not be checked again until the one-year timepoint (20, 21). Clinically, the status of free carnitine is considered when assessing for hypocarnitinemia (13). In addition, an potential indication of low carnitine in patients undergoing MAD can be elevated plasma triglycerides (22). Children on ketogenic diets with carnitine deficiency are typically given carnitine supplementation (18, 23) and some ketogenic diet centers start their pediatric patients on carnitine supplements prophylactically (20). Currently, there is minimal clinical guidance for adult carnitine monitoring or supplementation, and carnitine is typically only prescribed if deficiency is identified (21).

The goal of our retrospective analysis study is to explore the relationship between carnitine and MAD in the adult epilepsy demographic. Here, we evaluate the correlations of plasma carnitine levels with various metabolic variables associated with MAD and hypothesize that low plasma carnitine is correlated with poor control of seizures, low levels of plasma ketone bodies, high triglycerides, and taking multiple ASMs.

## 2 Materials and methods

### 2.1 Patient selection

Fifty-eight patients were retrospectively analyzed from the database of patients with epilepsy who have been prescribed MAD therapy at the UW Health Adult Neurology Ketogenic Diet Therapy Clinic. The patients’ data were extracted from the attending physician’s progress notes, results from lab measurements, and patient seizure diaries that ranged from June 2016 to June 2021. Inclusion criteria selected for patients with a diagnosis of epilepsy, an average of at least 2 seizures per month over the preceding 3 months prior to starting MAD, at least 18 years of age at the time of enrollment, having baseline carnitine lab measurement prior to starting MAD, and at least one carnitine lab measurement at a follow-up timepoint. Additionally, patients must follow MAD for at least 3 months, with some continuing MAD for over 2 years. Our study only included and analyzed data of follow-ups where patients were actively undergoing MAD therapy. Patients were requested to keep a stable medication regimen prior to starting and for at least 3 months of being on MAD.

### 2.2 Timeline and variables

All patient variables are tracked from the start of the diet to their current follow-up dates. The timepoints include baseline (prior to starting MAD) measurements, 3-9-month follow-up measurements, 1-2-year follow-up measurements, and 2+ year follow-up measurements. The variables selected for this study include patient characteristics, lab measurements, medications, and supplements. The patient characteristics include age, sex, weight, and body mass index (BMI). Lab measurements include total and free carnitine, fasting triglycerides, and beta-hydroxybutyrate (BHB). Medications and supplements include number of ASM, carnitine supplements, and valproic acid use. Additionally, patients were asked to track their seizure count and severity.

A value less than 25 μmol/L was considered low for free carnitine levels. Any value equal to or greater than 25 μmol/L was classified normal free carnitine. The cutoff for positive BHB status was 0.3 mmol/L or greater. Anything below 0.3 mmol/L was considered negative. To obtain the values for the average number of seizures per week, this calculation was achieved by tallying all the patients’ recorded seizures in the past 12 weeks and dividing the total number by 12. Seizure severity was recorded based on the patient’s own report.

### 2.3 Statistical methods

Demographics and characteristics of patients at baseline between the low carnitine status and the normal carnitine status were compared using Student’s t tests or chi-square tests. Generalized linear mixed effects models were used to compare the low carnitine status with the normal carnitine status on percent change from previous timepoint in seizure frequency, seizure severity, BHB level, number of side effects, fasting triglyceride, weight and BMI, where identity links were used for normally distributed outcome. Generalized linear mixed effects models were also used to compare the low carnitine status with the normal carnitine status on the number of ASMs, where a canonical log link function was used for Poisson distributed outcome. Because the model utilized was technically a generalized estimating equation (GEE) approach, the issue of over-overdispersion was automatically accounted for, since the variances were empirically estimated, rather than under the constraints of the Poisson distribution. All *p*-values were considered as significant if <0.05. All analyses were done by Statistical Analysis System (SAS) version 9.4, NC, Cary.

## 3 Results

### 3.1 Participant demographics

Table 1 summarizes the participant demographics at baseline prior to the start of MAD. There was a total of 58 patients with epilepsy undergoing diet therapy. Of the 58 patients, 11 patients (19%) presented with low free carnitine levels at baseline and 47 (81%) patients presented with normal carnitine levels at baseline. There were 40 female participants and 18 male participants ranging from age 19 to 68. At baseline, the average age of the patients was 38.94 ± 14.55, the average BHB level was 0.16 ± 0.08 mmol/L (negative, as expected prior to starting MAD), the average fasting triglyceride level was 102.95 ± 76.03, average body weight was 80.5 ± 23.18 kg, and average number of seizures per week was 2.68 ± 3.15. Four patients (6.90%) were taking no ASMs, 11 patients (18.96%) were on monotherapy with one ASM, while 43 patients (74.14%) were on polytherapy of two or more ASMs. Across all characteristics, there were no significant differences observed between patients with low and normal free carnitine levels at baseline.

**Table 1:** Demographics for patients at baseline.

		Free carnitine levels		
Characteristics prior to ketogenic diet therapy	All Patients (*n* = 58)	Low (*n* = 11)	Normal (*n* = 47)	*P*-value
Age (years), mea*n* ± SD	38.94 ± 14.55	36.78 ± 11.21	39.45 ± 15.29	0.589
BHB (mmol/L), mea*n* ± SD	0.16 ± 0.08	0.17 ± 0.08	0.15 ± 0.09	0.65
Triglycerides (mg/dL), mea*n* ± SD	102.95 ± 76.03	96.55 ± 57.54	104.48 ± 80.29	0.759
Body weight (kg), mea*n* ± SD	80.5 ± 23.18	71.52 ± 20.68	82.6 ± 23.43	0.155
Number of seizures per week, mea*n* ± SD	2.68 ± 3.15	1.75 ± 2.97	2.89 ± 3.19	0.283
Seizure severity change, mea*n* ± SD	–	–	–	
Sex				
Female # (%)	40 (69)	8 (73)	32 (68)	0.765
Male # (%)	18 (31)	3 (27)	15 (32)	
# of ASMs				
0 ASM (%)	4 (7)	2 (18)	2 (4)	0.231
1 ASM (%)	11 (19)	3 (27)	8 (17)	
2 ASMs (%)	28 (48)	3 (27)	25 (53)	
3 ASMs (%)	13 (22)	2 (18)	11 (23)	
4 ASMs (%)	2 (3)	1 (9)	1 (2)	

### 3.2 Carnitine deficiency across the course of diet therapy

Of the 47 patients with a normal free carnitine level at baseline prior to starting MAD, 18 (38.3%) patients developed free carnitine deficiency during the course of diet therapy. Within this group, 10 (55.6%) patients developed low free carnitine during the first follow-up (3 to 9 months) timepoint. Five (27.8%) of the 18 patients developed low free carnitine during the second follow-up (1 to 2 years) timepoint. Lastly, 3 (16.6%) patients developed hypocarnitinemia at the last timepoint follow-up (2+ years).

### 3.3 The effect of carnitine deficiency on diet efficacy and clinical variables

Table 2 details the frequency of participants within each clinical variable or measure group. Table 3 illustrates summary findings of group differences in percent change from previous timepoints for each variable and measure. Here, a generalized linear mixed effects model was utilized to adjust for repeated measures within the same subjects to compare between low free carnitine and normal free carnitine groups.

**Table 2:** Frequency of patients in each timepoint assessed for each variable.

	Group		Baseline	Timepoint 1 (~ 3–9 months of MAD)	Timepoint 2 (~ 1–2 years of MAD)	Timepoint 3 (~ 2 + of MAD)
Free carnitine	Low level (<25 μmol/L)	# (%)	11 (19)	16 (36)	8 (17)	5 (23)
	Normal level (≥25 μmol/L)	# (%)	47 (81)	28 (64)	38 (83)	17 (77)
# of seizures per week	Reduced/no seizures	# (%)	–	32 (71)	28 (61)	13 (62)
	No change/more seizures	# (%)	–	13 (29)	18 (39)	8 (38)
Seizures severity	Less severe/no seizures	# (%)	–	35 (80)	40 (85)	19 (86)
	No change/more severe	# (%)	–	9 (20)	7 (15)	3 (14)
BHB status	Positive	# (%)	–	35 (80)	27 (61)	16 (76)
	Negative	# (%)	–	9 (20)	17 (39)	5 (24)
# of ASMs	0 ASM	# (%)	4 (7)	5 (11)	3 (6)	2 (9)
	1 ASM	# (%)	11 (19)	10 (23)	12 (26)	6 (27)
	2 ASMs	# (%)	28 (48)	15 (34)	19 (40)	8 (36)
	3 ASMs	# (%)	13 (22)	13 (30)	10 (21)	4 (18)
	4 ASMs	# (%)	2 (3)	1 (2)	3 (6)	2 (9)
Valproic acid use	No	# (%)	51 (88)	53 (91)	53 (91)	54 (93)
	Yes	# (%)	7 (12)	5 (9)	5 (9)	4 (7)
Body mass index (BMI)	Underweight (≤ 18.5 kg/m2)	# (%)	4 (7)	1 (2)	1 (2)	0 (0)
	Normal (18.5─25 kg/m2)	# (%)	10 (17)	16 (36)	18 (38)	10 (48)
	Overweight (25─30 kg/m2)	# (%)	24 (41)	15 (34)	16 (34)	5 (24)
	Obesity class I (30─35 kg/m2)	# (%)	12 (21)	7 (16)	8 (17)	3 (14)
	Obesity class II (35─40 kg/m2)	# (%)	4 (7)	5 (11)	3 (6)	1 (5)
	Obesity class III (≥ 40 kg/m2)	# (%)	4 (7)	0 (0)	1 (2)	2 (10)

# of seizures and seizure severity reduction, increase, and no change is determined by comparison to previous timepoint.

**Table 3:** Group differences in % change from previous timepoint including timepoints 1, 2, and 3 (main effects for the group).

	Free carnitine		
Outcomes	Low level (< 25 μmol/L)	Normal level (≥ 25 μmol/L)	*P*-value
% Δ Number of seizures; mea*n* ± SD			
Baseline			
Timepoint 1	−16.82 ± 96.56	−42.51 ± 41.63	0.1637
Timepoint 2	67.68 ± 117.22	−10.68 ± 45.32	0.0372
Timepoint 3	−20.82 ± 56.75	−18.81 ± 41.45	0.8801
% Δ BHB Levels; mea*n* ± SD			
Baseline			
Timepoint 1	512.78 ± 269.93	371.13 ± 296.23	0.2221
Timepoint 2	−84.05 ± 2.97	50.33 ± 303.46	0.5064
Timepoint 3	208.67 ± 324.61	−10.72 ± 59.99	0.1746
% Δ Triglycerides; mea*n* ± SD			
Baseline			
Timepoint 1	36.56 ± 82.81	−1.37 ± 48.59	0.0386
Timepoint 2	−30.96 ± 58.74	−4.86 ± 35.69	0.4920
Timepoint 3	15.21 ± 38.32	9.24 ± 42.65	0.8347
% Δ Body mass index; mea*n* ± SD			
Baseline			
Timepoint 2	−6.18 ± 6.51	−5.55 ± 8.09	0.9734
Timepoint 2	−3.78 ± 2.94	−1.7 ± 5.7	0.6938
Timepoint 3	3.98 ± 9	1.84 ± 6.33	0.4624
# of ASM; mea*n* ± SD			
Baseline	1.73 ± 1.27	2.02 ± 0.82	0.5829
Timepoint 1	1.88 ± 1.09	1.89 ± 1.03	0.9544
Timepoint 2	1.25 ± 0.71	2.13 ± 0.99	0.1448
Timepoint 3	2 ± 0.71	1.88 ± 1.22	0.8593
% Seizure severity Δ; mea*n* ± SD			
Baseline			
Timepoint 1	0.25 ± 0.45	0.18 ± 0.39	0.7024
Timepoint 2	0.25 ± 0.46	0.13 ± 0.34	0.5874
Timepoint 3	0 ± 0	0.18 ± 0.39	1.000

ASM, anti-seizure medication depicted as the number of medications prescribed as opposed to the percent change.

*P-values are derived from generalized mixed effect models.

Δ = change.

**Figure 1:**
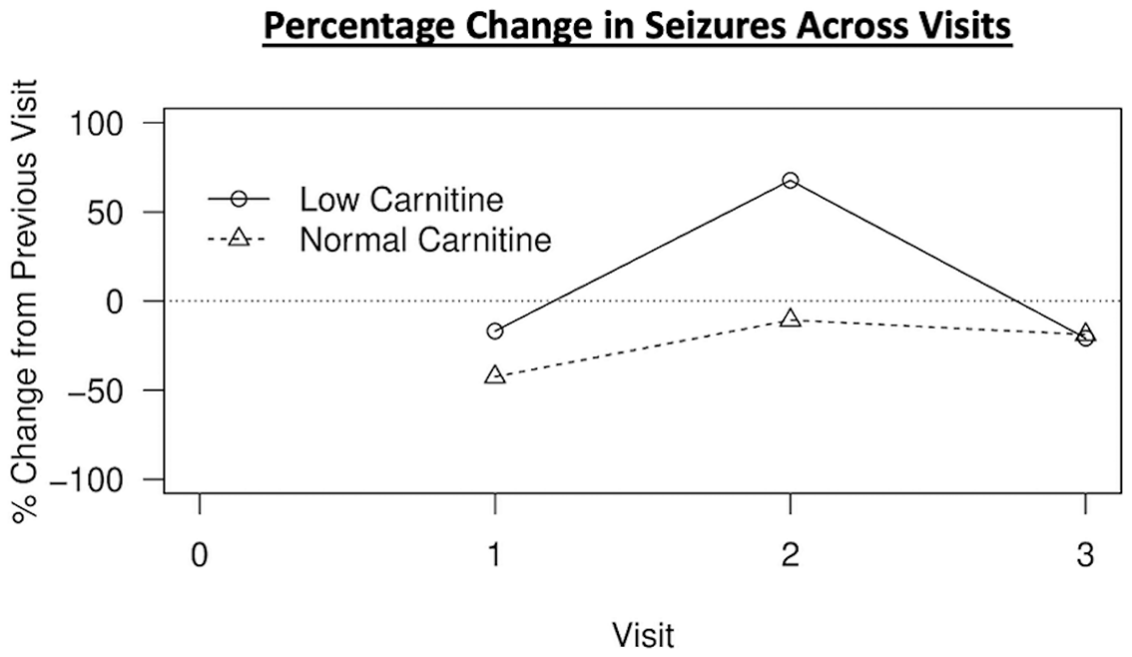
Percent change in seizures from previous timepoint over time during MAD therapy.

To investigate the effect of hypocarnitinemia on MAD efficacy, we assessed the percentage change in the number of seizures between patients with low free carnitine and normal free carnitine for each follow up timepoint. Our results revealed no statistical differences at the first timepoint (3 to 9 months). Interestingly, the low free carnitine group illustrated a seizure increase of 67.68% at the second timepoint (1 to 2 years) and is significantly different (*p* = 0.0372) compared to the normal carnitine group, which showed a seizure reduction of 10.68%. The third timepoint (2+ years) revealed no significant differences between the two carnitine profiles. Figure 1 demonstrates the trend of % seizure change over the timepoints comparing the normal free carnitine group with the low free carnitine group. From this figure, we can visualize that for timepoints 1 and 2, normal free carnitine patients experience greater % change in seizure reduction on MAD compared to low free carnitine patients. This difference is significant and apparent during the second timepoint where low carnitine patients experienced an increase in % seizure change as opposed to the % seizure reduction expected from undergoing MAD therapy. Additionally, it is important to point out that patients in the normal carnitine group continued to experience a reduction in the number of seizures at each of 3 timepoints.

When examining BHB (blood ketone) levels, no significant differences were observed at any timepoint between low and normal free carnitine groups. When inspecting fasting triglyceride levels at the first timepoint after initiating MAD therapy, low free carnitine showcased a 36.56% triglyceride level increase that was significantly different (*p* = 0.0386) compared to 1.37% triglyceride level decrease in the normal free carnitine group. Later timepoints revealed no significant triglyceride differences between the low and normal free carnitine groups. Moreover, measures of BMI, changes in seizure severity, and number of ASMs demonstrated no significant differences across all timepoints between the carnitine profiles.

## 4 Discussion

Overall, our results revealed that 38.3% of adult patients with epilepsy following MAD experienced low free carnitine at some point through the course of diet therapy. Within that, the majority of our patients developed hypocarnitinemia within the first 3–9 months of initiating MAD; however, further timepoint measurements demonstrates that hypocarnitinemia may arise at any time during the course of the diet, even after 2+ years. Our analysis highlighted significant differences in % seizure changes in the second (1–2 year) timepoint where the low free carnitine group demonstrated 67.68% seizure increase from the previous timepoint (3–9 months) when compared to the normal free carnitine group, which had a seizure reduction of 10.68%. Furthermore, examination of fasting triglyceride levels during the first timepoint follow up (3–9 months) illustrated an increase in triglyceride level by 36.56%, which was significantly difference from the 1.37% triglyceride decrease in the normal free carnitine group.

While we observed no differences in the change in seizure severity between patients with hypocarnitinemia and normal free carnitine levels, Table 2 illustrates that 80% of our patients undergoing MAD reported less severe seizures or complete seizure freedom at the first timepoint, 85% at the second timepoint, and 86% at the third timepoint. This uncovers that regardless of carnitine levels, MAD may be a powerful option to reduce overall seizure severity in adults with epilepsy.

The International Recommendations for the Management of Adults Treated With Ketogenic Diet Therapies noted that the majority of adult clinics check carnitine levels at baseline and at 1 year (21). Our findings showed that more than 1 in 3 adults on MAD developed carnitine deficiency while on diet therapy. More than half of those adults developed carnitine deficiency within the first 3–9 months. Adults in the low carnitine group experienced an increase in seizures during the 1–2-year timepoint while adults with normal carnitine continued to reap the benefits of ongoing seizure reduction. It may be pertinent for clinicians to check carnitine levels with other routine diet therapy lab monitoring (at 3 months, 6 months, then every 6 months) since patients are often asymptomatic, and carnitine deficiency may go undetected if it is not checked. If seizures increase on diet therapy, patients may discontinue ketogenic diet therapy prematurely due to perceived lack of efficacy when the issue may be low carnitine. Routine lab monitoring may also be a helpful marker of compliance when a carnitine supplement, such as levocarnitine, is prescribed and it can help clinicians adjust the prescription to optimize serum levels.

Interestingly, we did not see an association between BHB levels with % seizure reduction. Some studies have found a correlation between BHB and seizure control (24, 25). However, other recent studies have reported that BHB was not able to predict seizure reduction in children reliably, but found that plasma acylcarnitine did (26). In our practice, patients anecdotally report lower ketone levels prior to identification of carnitine deficiency. Analysis showed no statistically significant difference in BHB between patients with low carnitine and those with normal carnitine levels. The BHB level used for analysis was drawn as part of fasting labs typically drawn in the morning. BHB is typically lower in the morning due to the dawn phenomenon, which causes an increase in blood glucose. The time of the lab draw may be why there was no statistically significant difference between groups and no association between BHB level and % seizure reduction. Future studies should examine BHB at various time points during the day using a home blood ketone meter. Since BHB may also vary from patient to patient, it would also be pertinent to look for changes in an individual patient’s BHB to see if average BHB changes when a patient develops carnitine deficiency.

While previous research has indicated polytherapy as a risk factor for hypocarnitinemia in patients with epilepsy (13, 15, 17, 19), our analysis demonstrates no statistically significant associations between taking multiple ASMs and hypocarnitinemia. In addition, a previous study identified 38% of their patients taking valproic acid and 24% taking carbamazepine had hypocarnitinemia. In our present study, only 2 patients (29%) had hypocarnitinemia out of the 7 patients taking valproic acid at baseline and 2 patients (50%) had hypocarnitinemia out of the 4 patients taking carbamazepine. However, it is important to note that in our study, all patients who were taking valproic acid and carbamazepine were on polytherapy. It would be noteworthy for future studies to track carnitine levels of all patients who are taking valproic acid or carbamazepine over the course of the ketogenic diet and observe whether hypocarnitinemia is more prevalently developed in these subsets of patients over the course of the diet.

Red meats are the richest food source of carnitine (27). Other food sources including dairy products, fish, poultry, avocado, and asparagus contain some carnitine, but much lower amounts (28). Since vegetarian food sources contain only small amounts of carnitine, patients on a vegetarian ketogenic diet are especially at increased risk for carnitine deficiency. Carnitine supplementation may be indicated for patients exhibiting hypocarnitinemia throughout the course of the diet.

Our study has limitations. A future prospective study with a greater number of participants that can provide adequate power to the analysis would be beneficial. Many of our patients were undergoing MAD throughout the course COVID-19 pandemic, which affected routine follow-up appointments and lab measurements. Naturally, our number of participants returning for follow-up appointments and lab draws decreased over the course of the study. Future studies on carnitine should be checked more routinely. Furthermore, the serum BHB level may vary depending on the time of day as BHB tends to be lower in the morning.

## Data availability statement

The raw data supporting the conclusions of this article will be made available by the authors, without undue reservation.

## Ethics statement

The requirement of ethical approval was waived by the University of Wisconsin Madison Institutional Review Board for the studies involving humans because this is a retrospective chart review project. The studies were conducted in accordance with the local legislation and institutional requirements. The ethics committee/institutional review board also waived the requirement of written informed consent for participation from the participants or the participants’ legal guardians/next of kin because Retrospective chart review project.

## Author contributions

DC: Conceptualization, Data curation, Formal analysis, Investigation, Methodology, Supervision, Validation, Visualization, Writing – original draft, Writing – review & editing. MR: Conceptualization, Formal analysis, Investigation, Methodology, Supervision, Writing – review & editing. KF: Conceptualization, Methodology, Resources, Supervision, Writing – review & editing. AW: Data curation, Writing – review & editing. DA: Data curation, Project administration, Resources, Writing – review & editing. ZL: Formal analysis, Writing – review & editing. ES: Formal analysis, Writing – review & editing. EF: Conceptualization, Data curation, Funding acquisition, Investigation, Methodology, Project administration, Resources, Supervision, Validation, Visualization, Writing – original draft, Writing – review & editing.
